# Unsupervised Approaches to Finding Outliers in Caption-Represented Images

**DOI:** 10.3390/e27070661

**Published:** 2025-06-20

**Authors:** Jakub Zaprzałka, Magdalena Topczewska

**Affiliations:** Faculty of Computer Science, Bialystok University of Technology, Wiejska 45A, 15-351 Bialystok, Poland; m.topczewska@pb.edu.pl

**Keywords:** unsupervised learning, data visualization, outliers, multidimensional scaling, agglomerative clustering

## Abstract

Both supervised and unsupervised machine learning algorithms are often based on regression to the mean. However, the mean can easily be biased by unevenly distributed data, i.e., outlier records. Batch normalization methods address this problem to some extent, but they also influence the data. In text-based data, the problem is even more pronounced, as distance distinctions between outlier records diminish with increasing dimensionality. The ultimate solution to achieving unbiased data is identifying the outliers. To address this issue, multidimensional scaling (MDS) and agglomerative-based techniques are proposed for detecting outlier records in text-based data. For both methods, two of the most common distance metrics are applied: Euclidean distance and cosine distance. Furthermore, in the MDS approach, both metric and non-metric versions of the algorithm are used, whereas in the agglomerative approach, the last-*p* and level cutoff techniques are applied. The methods are also compared with a raw-data-based method, which selects the most distant element from the others based on a given distance metric. Experiments were conducted on overlapping subsets of a dataset containing roughly 2000 records of descriptive image captions. The algorithms were also compared in terms of efficiency with a proposed algorithm and evaluated through human judgment based on the described images. Unsurprisingly, the cosine distance turned out to be the most effective distance metric. The metric-MDS-based algorithm appeared to outperform the others based on human evaluation. The presented algorithms successfully identified outlier records.

## 1. Introduction

Nowadays, powerful image generation models based on natural language processing, e.g., DALLE [1], PaLM [2], Stable Diffusion [3], or Gemini [4], are trained on large amounts of collected and labeled images. An accurate and consistent labeling process is a crucial part of the initial stage of data preparation—an essential step for training the models effectively. Even a small number of incorrectly described objects can lead to inaccurate or completely failed results. Human labelers are employed to describe images, and therefore, the need to improve these descriptions may arise.

In this article, we consider images with atypical descriptions as outliers within the dataset. Their unusual descriptions may result from uncommon objects in the image or incorrect word choices used to describe the elements.

Outlier elements can often cause machine learning algorithms to fail, as they do not conform to the same bounds as the majority of records in the dataset [5]. However unrelated such records may appear, they do not necessarily lack relevance to the problem at hand. In fact, these outlier elements may provide exactly the information being sought. Nevertheless, they can introduce anomalies in machine learning algorithms. With awareness of these elements in the dataset and the ability to identify them, such anomalies can be more effectively detected, diagnosed, prevented, or explained during the final analysis.

Although identifying outliers in simple, low-dimensional datasets is primarily a statistical task, in the case of multidimensional, multicategorical datasets—such as tag-labeled images—the problem becomes significantly more complex.

The scope of this article is to identify outlier elements in multidimensional, multicategorical data using a variety of methods. These methods are then compared in terms of their functionality, performance, and results.

Outlier records are selected to identify images that require additional attention during data preparation. Such records should undergo further analysis to determine the appropriate handling strategy. Some may require corrections to their descriptions, while others may need to be removed or supplemented by similar records to improve dataset balance. In rare cases, they may even be safely ignored. However, the handling of detected outlier records—unlike their detection—is beyond the scope of this article.

## 2. Related Research

Outlier detection in textual data has long been recognized as a key challenge for maintaining dataset quality, particularly in the context of large-scale language model (LLM) training [6,7]. As LLMs grow increasingly reliant on vast and noisy corpora, the ability to filter out anomalous or mislabeled samples becomes critical to model performance and fairness. This issue extends to the fine-tuning phase, where even small quantities of low-quality data can disproportionately affect model behavior [8,9].

With the rise of multimodal models [10,11], accurate identification of mismatched or misleading image-caption pairs becomes crucial for the construction of robust training datasets. Multimodal outlier detection techniques often rely on cross-modal embeddings or similarity scoring between textual and visual modalities. Prior work has explored methods such as contrastive pretraining, joint embedding learning, and unsupervised anomaly detection to address this challenge [12,13].

Although existing approaches have proven effective to varying degrees, a persistent limitation in large-scale datasets is the presence of ambiguous or borderline outliers—data points that are flagged by some methods but not others. This raises questions about method reliability and applicability across domains and data types.

Recent methods have explored generative approaches to improve outlier detection in high-dimensional data. GSAAL (Generative Subspace Adversarial Active Learning) employs multiple adversarial discriminators to identify informative subspaces before ensemble detection, addressing both the curse of dimensionality and multiple-view issues [14]. Building on similar principles, ASG (Adversarial Subspace Generation) leverages a generative model combined with new theoretical insights to discover optimal subspaces efficiently—significantly enhancing unsupervised outlier detection performance [15]. In the text domain, unsupervised clustering and density-based techniques (e.g., GMM, Isolation Forest) have shown strong results in filtering noisy multilingual corpora, validating the efficacy of language-independent preprocessing methods [7].

To address these concerns, this work contributes in two primary ways. First, it evaluates and compares existing outlier detection techniques for caption-based datasets using both semantic (e.g., metric MDS) and structure-based (e.g., Agglomerative Outlier Selection) approaches. Second, it proposes a novel, dataset-aware, non-parametric evaluation metric for method comparison. The proposed Agreement-Based Evaluation Metric offers a fair measure of how consistently a method agrees with others in identifying outliers. It allows dataset-specific ranking of proposed outliers without assuming any ground truth labels—an essential property for unsupervised settings.

This perspective acknowledges that not all flagged records are truly erroneous, and those selected less frequently across methods may still contain valuable information. The proposed methodology supports nuanced decisions about whether to retain or discard such records during dataset curation.

## 3. Methods

The following section presents the methods employed to detect outliers in the dataset. Several approaches were explored, each leveraging different representations and assumptions about the data. These include classical dimensionality reduction techniques such as Metric and Non-Metric Multidimensional Scaling (MDS), real-data-based distance filtering, and the custom Agglomerative Outlier Selection (AOS) based on hierarchical clustering. Each method was applied using either cosine or Euclidean distance metrics, both of which are discussed in detail.

### 3.1. Dataset Description

The dataset used in this study consists of image-caption pairs. The analysis focuses solely on the captions, which are transformed into binary bag-of-words vectors. Each vector encodes the presence or absence of words across a predefined vocabulary, forming a sparse, high-dimensional binary sparse matrix (i.e., filled with zeros)—a structure typical for text-based datasets.

The bag-of-words (BoW) model [16] is a simple yet effective method for representing textual data. It disregards grammar and word order, focusing solely on the occurrence of individual words. In this study, the BoW representation is binary: for each caption, a vector of fixed vocabulary size is generated, where each entry is set to 1 if the corresponding word is present in the caption, and 0 otherwise. This representation enables the use of standard distance metrics and dimensionality reduction techniques without requiring semantic embeddings.

The original images serve only as visual representations and are not used in any computational step. For consistency across experiments and to reduce computational overhead, a fixed subset of 500 records is used in all evaluations.

### 3.2. Distance Metrics

Two distance metrics are employed in this study to construct pairwise distance matrices: cosine distance and Euclidean distance. Cosine distance is particularly effective for high-dimensional, sparse binary data such as bag-of-words vectors, as it captures angular dissimilarity between feature vectors and is robust to variations in vector magnitude. This makes it a widely used similarity measure in text mining and natural language processing tasks [17,18]. In contrast, Euclidean distance is a more traditional choice for continuous numerical data and is included here for comparison purposes.

A cosine-based distance matrix is computed by measuring pairwise distances between all caption vectors. From this matrix, the minimum distance from each data record to any other is determined. The distribution of these minimum distances is visualized as a histogram, and a cutoff threshold—typically set at the 99th percentile—is used to identify potential outliers. Records with minimum distances exceeding this threshold are flagged as outlier candidates [19]. The exact threshold value, along with other key parameters used across all methods, is detailed in Section 3.5.

To enable comparative analysis of detection robustness, several outlier detection methods are evaluated using both cosine and Euclidean distances, allowing the influence of the distance metric choice to be systematically assessed.

### 3.3. MDS Based Solution

This solution requires the use of Multidimensional Scaling (MDS) [20]—a machine learning technique used to represent multidimensional data on a two-dimensional surface, approximating distances such that the distances between records best reflect the original ones. MDS may help refine approximations and distinguish outlier images more effectively. The algorithm is very similar to the solution presented above. However, instead of using the cosine-similarity-based distance matrix as the primary input, the output of MDS—the locations of records on a two-dimensional plane—is used to prepare a new distance matrix, which then serves as the basis for the main algorithm. As input for MDS, the cosine-similarity-based distance matrix is used.

Multidimensional Scaling can be computed in both metric and non-metric forms. Both variants employ the SMACOF algorithm [20]—however, the stress calculation method differs slightly. The metric variant uses a direct approach to the target function and minimizes its squared loss.

(1) $$\mathrm{stress}=L\left(\hat{d_{ij}}\right)=\sqrt{\frac{\sum_{i<j}{\left(\hat{d_{ij}}-f\left(d_{ij}\right)\right)}^{2}}{\sum d_{ij}^{2}}}$$

The non-metric method’s approach, also called Kruskal’s non-metric MDS [21], works slightly differently, as it minimizes the *stress-1* function for the given distance matrix.

(2) $$\mathrm{stress}-1\left(\hat{d_{ij}},d_{ij}^{*}\right)=\sqrt{\frac{\sum_{i<j}{\left(\hat{d_{ij}}-d_{ij}^{*}\right)}^{2}}{\sum d_{ij}^{2}}}$$

In this case, $d_{ij}$ are the dissimilarities, and $d_{ij}^{*}$ are the disparities. Dissimilarities represent the observed ordinal distances between objects, while disparities are their monotonic transformations, used to best preserve the rank order in the Euclidean embedding. The non-metric MDS algorithm adjusts the disparities to minimize stress while maintaining the ordinal structure of the dissimilarities.

To support clarity and reproducibility, Algorithm 1 outlines the full procedure for outlier selection using MDS-based dimensionality reduction. The algorithm accommodates both metric and non-metric variants, with the primary distinction being the construction of the input matrix: metric MDS requires a distance matrix computed using a predefined metric (e.g., Euclidean or cosine), while non-metric MDS operates on a dissimilarity matrix that preserves the rank order of pairwise relationships. Both approaches proceed by embedding the data into a lower-dimensional space, followed by identifying records whose minimum distances to any other embedded point exceed a selected percentile threshold.

### 3.4. Agglomerative Clustering Driven Approach

Hierarchical clustering methods perform clustering in the most unsupervised manner among all clustering techniques [22]. Similar to MDS, the distance between records can be calculated using either the Euclidean or cosine metric. Instead of initially finding cluster centers and iteratively recalculating them to minimize the distance between centers and records—a global approach—hierarchical methods start by locally joining the two closest records in the dataset and creating a cluster center at the mean point between them.
**Algorithm 1** MDS-Based Outlier Selection**Require**: **X**: binary input matrix (BoW)**Require**: *d*: target embedding dimensionality**Require**: *k*: outlier cutoff percentile**Require**: *T*: MDS type (*metric* or *non-metric*)**Require**: *M* (optional): distance metric (for metric MDS)**Ensure**: **O**: indices of detected outliers  1: **if** *T* is metric **then**  2:     Compute pairwise distance matrix *D* using metric *M*  3: **else**  4:     Compute dissimilarity matrix *D* using rank-based method  5: **end if**  6: Apply MDS of type *T* to *D* to obtain embedding Y  7: For each embedded point, compute its minimum distance to others  8: Determine threshold at the *k*-th percentile of those distances  9: Identify records with minimum distances exceeding the threshold10: **return** O: indices of selected outliers

This process creates a dendrogram of connections, with the distance between clusters indicated by the dendrogram level. The hierarchical dendrogram can be either cut off at a certain level (level cutoff method) or limited to the last *p* merged clusters (last-*p* cutoff method), thereby creating clusters.

The dendrogram can also be analyzed so that clusters—mainly singleton clusters—connected at the end are treated as potential outlier records [23]. The potential outliers are then sorted in reverse order of the level at which they were joined in the dendrogram, and the first k=1% of the dataset’s records are selected as outliers. The method of cutting off the dendrogram and extracting outliers [23] is hereafter referred to as Agglomerative Outlier Selection (AOS).

Depending on the dendrogram cutoff method used to select unjoined singleton clusters, the number of outliers may vary. With the level cutoff method, all records joined at the same dendrogram level are selected as outliers, whereas with the last-*p* cutoff method, the last *p* merges are treated as potential outliers.

To illustrate the full procedure of the Agglomerative Outlier Selection approach, Algorithm 2 presents its implementation in detail. The algorithm applies agglomerative clustering to the distance matrix and selects outliers based on one of two cutoff strategies: level cutoff, which retrieves all singleton clusters joined at a specific dendrogram height, and last-*p* cutoff, which extracts records involved in the last *p* linkage operations. These cutoff strategies allow for flexible control over the number and type of outliers extracted, depending on the desired granularity and the structure of the dendrogram.
**Algorithm 2** Agglomerative Outlier Selection (AOS)**Require**: Distance matrix D, cutoff method cutoff_type, cutoff parameter param**Ensure**: Set of selected outlier indices Outliers  1: Z←AgglomerativeLinkage(D)            ▹ Run agglomerative clustering and record linkage matrix  2: **if** 
cutoff_type=“level”
** then**  3:     threshold←param  4:     Outliers←{recordsinsingletonclustersjoinedatthreshold}  5: **else if** 
cutoff_type=“last_p”
 **then**  6:     p←param  7:     last_p_merges←lastprowsofZ  8:     Outliers←uniquerecordindicesinvolvedinlast_p_merges  9: **else**10:     **raise** Error: Unknown cutoff method11: **end if**12: **return** 
Outliers

In this way, agglomerative clustering is used to identify outlier images in the proposed solution.

### 3.5. Hyperparameter Selection

The choice of hyperparameters in the proposed methods was made empirically or computationally, depending on the context. For both the raw-distance and MDS-based approaches, the cutoff threshold was set at the 99th percentile of the minimal distance distribution—equivalent to selecting the top k=1% of records with the highest minimum distance. This value was chosen purely empirically to demonstrate the effectiveness of the method and was found to consistently yield the most meaningful outlier selections.

For the agglomerative clustering approach, two cutoff strategies were used: the level cutoff and the last-*p* cutoff. In both cases, the cutoff parameter (i.e., the dendrogram level or the number of last merges *p*) was chosen computationally to ensure a fair comparison between methods. Specifically, the first level or *p* value was selected such that the number of detected outliers was greater than or equal to the number of outliers identified by the MDS-based method.

This consistent approach to hyperparameter selection allowed for an approximately balanced outlier count across techniques, facilitating a more meaningful evaluation of their performance.

### 3.6. Comparison and Evaluation

As previously stated, the task of identifying outlier records was approached using multiple techniques. Regardless of the method, either the metric or non-metric MDS algorithm was always employed to represent the data in a two-dimensional space. In the results obtained through MDS-driven approaches (see Section 3.6.1), the same variant of MDS used to produce the results was also used for visualization. In contrast, for the AOS-driven approaches (see Section 3.6.2), visualization was performed using metric MDS based on the cosine distance matrix.

To evaluate performance, the dataset was examined in terms of subsets of varying sizes. These subsets were constructed hierarchically—each larger subset includes all the records from the smaller subsets. The performance and runtime statistics of the MDS algorithm for each subset size are summarized in Table 1.

The records—described by their associated tags—can be visualized as points in two-dimensional space. These visualizations, generated after applying the MDS algorithm, were presented as plots to support qualitative evaluation.

The following sections detail the results of experiments using the methods introduced in Section 3. For technical reasons, visualizations are shown only for the subset of 500 records. A selection of visualizations for other subsets (see Table 1) will be included in the Appendix A.

#### 3.6.1. MDS-Driven Solution

The MDS-based solution was obtained using the approaches described in Section 3.3. The detected outlier records are presented in comparison to the real-data-based method.

The MDS-based solution allows for intuitive visual detection of outliers in the reduced-dimensional space. Although the real-data-based method selects records based on their original distance profiles, these selections may not align perfectly with patterns observed after dimensionality reduction. However, some records—such as the one with ID 54—are consistently identified as outliers by both methods, underscoring their distinctiveness within the dataset.

##### Cosine Distance

**Figure 1:**
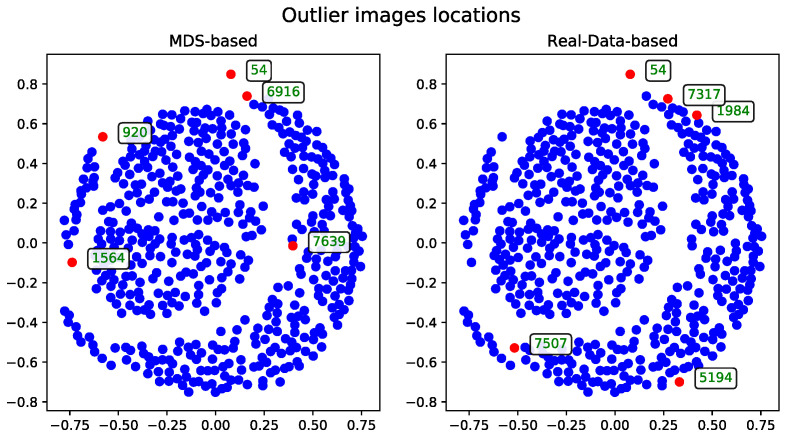
Outlier records (marked with red) selected by metric MDS for a subset of 500 images using the cosine distance matrix. The displayed IDs correspond to the same records across all method visualization plots. Similar plots for other subsets will be included in the Appendix A.

The MDS algorithm was executed using the cosine distance matrix [17]. The outliers identified by this method, alongside those detected using the real-data-based approach, are presented in Figure 1.

##### Metric Versus Non-Metric

**Figure 2:**
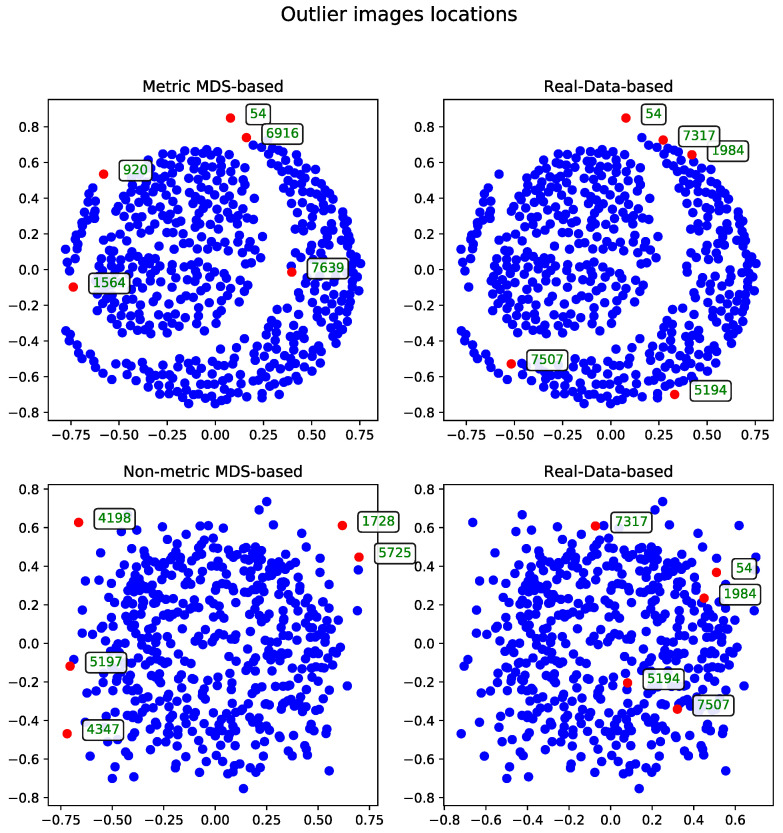
Comparison of outlier records (marked with red) selected by metric and non-metric MDS for a subset of 500 images using the cosine distance matrix. The displayed IDs correspond to the same records across all method visualization plots.

The data can be processed using either the metric or non-metric version of the Multidimensional Scaling algorithm. The theoretical differences between the two are discussed in Section 3.3, while the practical differences are illustrated in Figure 2.

For comparison, the real-data-based solution is shown alongside both visualizations—one generated using metric MDS and the other using non-metric MDS. The non-metric MDS produces a less structured representation, often identifying outliers in a seemingly arbitrary manner. This behavior stems from its reliance on rank-based distance preservation rather than absolute distances. In contrast, the outliers detected by the real-data-based method show much stronger alignment with the metric MDS projection. Notably, all outliers selected by the real-data-based method are located within the lower crescent-shaped cluster, whereas the metric MDS highlights additional patterns and anomalies, offering a complementary perspective on the dataset.

##### Euclidean Distance

**Figure 3:**
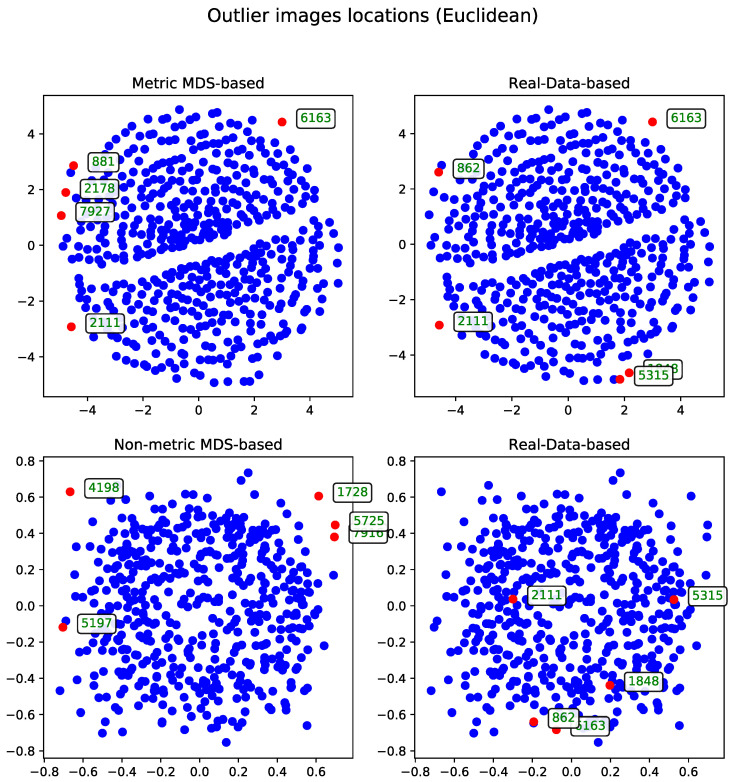
Comparison of outlier records (marked with red) selected by metric and non-metric MDS for a subset of 500 images using the Euclidean distance matrix. The displayed IDs correspond to the same records across all method visualization plots.

The second approach to the MDS problem utilized the Euclidean distance matrix. Results from both the metric and non-metric MDS algorithms are presented for this configuration. Figure 3 illustrates the outcomes of both methods—in a manner analogous to the comparison in Section Metric Versus Non-Metric.

#### 3.6.2. Agglomerative-Clustering-Based Solution–Agglomerative Outlier Selection

The dendrogram produced by the agglomerative clustering method serves as the foundation for identifying outlier records in the data. This approach can be applied using different affinity metrics and linkage algorithms.

##### Euclidean Distance

**Figure 4:**
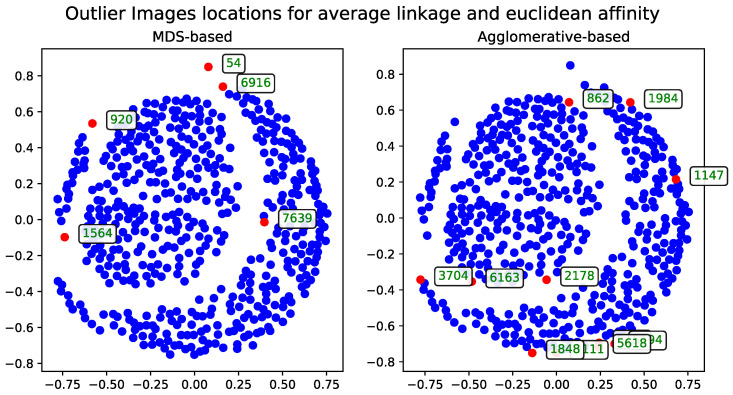
The comparison of the outlier records (marked with red) selected by the AOS algorithm with a level cutoff for a subset of 500 images based on the Euclidean distance matrix. The displayed IDs correspond to the same records across all method visualization plots.

**Figure 5:**
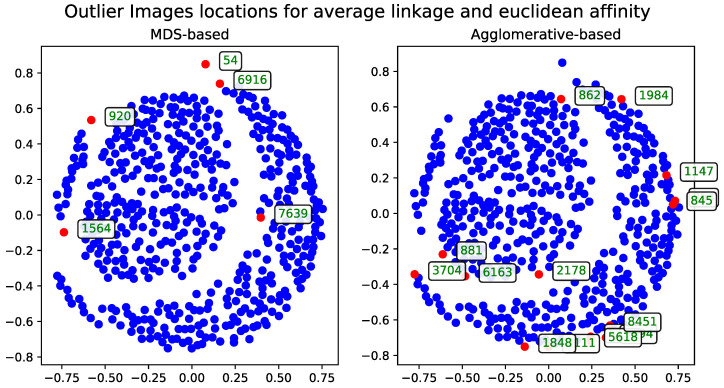
The comparison of the outlier records (marked with red) selected by the AOS algorithm with last-*p* cutoff for a subset of 500 images based on the Euclidean distance matrix. The displayed IDs correspond to the same records across all method visualization plots.

The results calculated with Euclidean distance were produced using two dendrogram cutoff methods—level (Figure 4) and last-*p* (Figure 5). Both methods are compared to the metric MDS approach based on the cosine distance matrix, which is also used to visualize the results.

The dendrogram cutoff parameters for both the level and last-*p* methods were chosen to ensure that the number of detected outliers is comparable to those found by the MDS-based approaches, as detailed in Section 3.5. This approach ensures the number of outliers is comparable across all methods.

##### Cosine Distance

**Figure 6:**
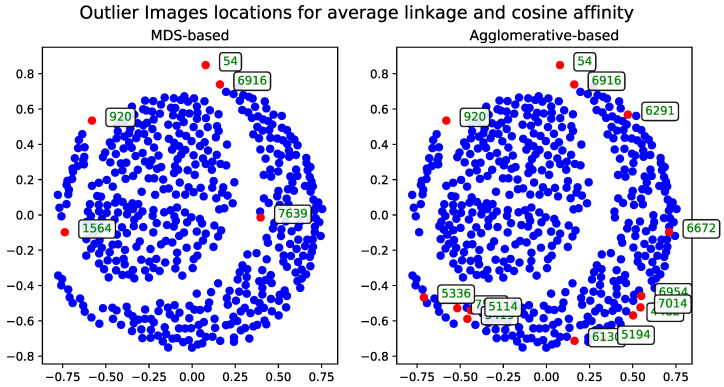
The comparison of the outlier records (marked with red) selected by the AOS algorithm with a level cutoff for a subset of 500 images based on the cosine distance matrix. The displayed IDs correspond to the same records across all method visualization plots.

**Figure 7:**
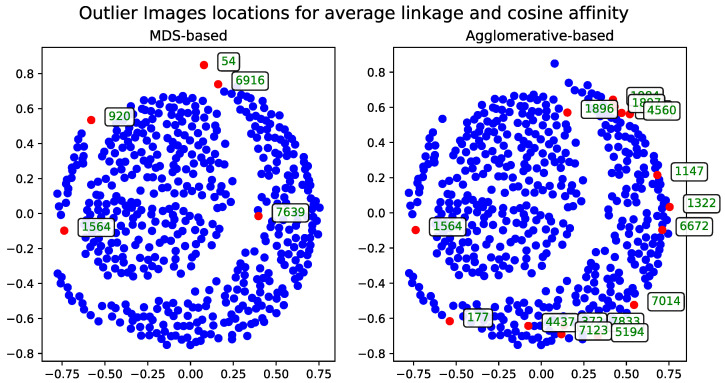
The comparison of the outlier records (marked with red) selected by the AOS algorithm with last-*p* cutoff for a subset of 500 images based on the cosine distance matrix. The displayed IDs correspond to the same records across all method visualization plots.

The agglomerative clustering dendrogram was also constructed using the cosine distance matrix. The AOS results obtained from this dendrogram were generated with two dendrogram cutoff methods—level (Figure 6) and last-*p* (Figure 7). Both methods are compared to the metric MDS approach based on the cosine distance matrix, which is likewise used for visualizing the results.

The dendrogram cutoff parameters follow the selection criteria detailed in Section 3.5.

## 4. Results and Practical Considerations

The outlier records identified by each method were gathered and are presented in the tables in this section. For practical reasons, all experiments that produced the results analyzed here were conducted only on the subset of 500 records. However, the methods can be safely applied to the other subsets as well. A selection of additional results obtained on other subset sizes is included in the Appendix A, and supports the conclusions presented here. A summary of the outliers and their corresponding IDs, categorized by method and distance metric, is presented in Table 2.

Table 2 and Table 3 use consistent color coding and font styles to visually indicate outliers selected by multiple methods, allowing quick identification of overlaps across different algorithms and distance metrics.

Some records were identified as outliers by multiple methods, while others were flagged by only one. These overlaps are visually represented in Table 2 using color and font style to highlight similarities between the selections. Not all methods returned the same number of outliers—particularly the AOS algorithm, whose output varied depending on the configuration. This variation is due to its dependence on the hierarchical structure of the dendrogram, where the number of selected records is influenced by the size and position of the clusters joined at the upper levels. Despite these differences, the total number of outliers selected by each method remains in the same order of magnitude, making the results comparable. The exact counts of outliers selected by each method and configuration are summarized in Table 4.

As shown in Table 4, the number of outliers selected by each method varies depending on both the algorithm and the chosen configuration. While the real-data-based method and both MDS variants consistently return five outliers—either by design or due to parameter selection—the AOS algorithm exhibits greater variability. This variability stems from its hierarchical nature, where the final selection is shaped by the dendrogram structure and the chosen cutoff strategy. The level-based cutoff typically selects fewer records than the last-*p* variant, reflecting their differing assumptions about cluster granularity. These differences are further discussed in the context of parameter influence and selection strategies in Section 3.5.

To further evaluate consistency among methods, each pair of algorithms was analyzed in terms of shared outlier selections. The analysis is summarized in two triangle-matrix-style tables. Table 3 lists the specific record IDs that were selected as outliers by both methods in each pair. In contrast, Table 5 presents the corresponding number of shared outliers per pair, offering a quantitative perspective on overlap.

Lastly, an outlier-oriented statistic was calculated for each method. This statistic served as the basis for constructing a metric for evaluating the outlier selection approaches. A higher value of the metric indicates that a greater number of outliers selected by a given method were also identified by other methods. The procedure for computing this evaluation metric is presented in Algorithm 3.
**Algorithm 3** ABEM: Agreement-Based Evaluation Metric for Outlier Selection Methods  1: $n_{m}\leftarrow \left|\mathrm{methods}\right|$                                                                                 ▹ Number of methods  2: $n_{o}\leftarrow \left|\mathrm{all}\_\mathrm{outliers}\right|$                       ▹ Total number of unique outliers across all methods  3: $M\leftarrow \mathrm{zero}\;\mathrm{matrix}\;\mathrm{of}\;\mathrm{size}\;n_{o}\times n_{m}$                ▹ Binary presence matrix: outlier × method  4: **for** m=1 to $n_{m}$ **do**  5:     **for** each o∈ outliers selected by method *m* **do**  6:         M[o,m]←1  7:     **end for**  8: **end for**  9: **for** i=1 to $n_{o}$ **do**                               ▹ Compute co-occurrence weights for each outlier10:     $w_{i}\leftarrow \sum_{j=1}^{n_{m}}M\left[i,j\right]$11: **end for**12: **for** m=1 to $n_{m}$ **do**                      ▹ Compute average agreement score for each method13:     $S_{m}\leftarrow \mathrm{mean}\;\mathrm{of}\;w_{i}\;\mathrm{for}\;\mathrm{all}\;i\;\mathrm{such}\;\mathrm{that}\;M\left[i,m\right]=1$14: **end for**15: **Return** sorted list of scores $S_{1},\ldots ,S_{n_{m}}$ in descending order

The Agreement-Based Evaluation Metric for Outlier Selection Methods (ABEM) captures the mean frequency with which the outliers selected by a given method also appear in the selections of other methods. As outlined in Algorithm 3, this is achieved by first constructing a binary matrix indicating which methods selected which outliers. The matrix is then weighted based on the number of methods that selected each outlier. Finally, for each method, the metric is computed as the average weight of its selected outliers. A higher value of this metric indicates stronger agreement between the evaluated method and the rest of the methods in the comparison set.

The results of applying the Agreement-Based Evaluation Metric for Outlier Selection Methods (ABEM) to the set of methods described in Section 3—based on experiments conducted on the subset of 500 records—are presented in Table 6. The table lists the mean ABEM values, providing a quantitative measure of how much each method’s selected outliers overlap with those selected by other methods. Higher values indicate greater agreement with the rest of the method set.

A value of ABEM close to 1 suggests that most outliers selected by the corresponding method were unique, i.e., not selected by any other method. This lack of overlap may indicate that the method captures a divergent notion of outliers, which could suggest lower practical reliability or efficiency. Conversely, higher ABEM values indicate greater agreement with other methods—meaning that outliers identified by the method were more frequently shared across the evaluation set. This consensus can be interpreted as a proxy for robustness, possibly pointing to a more effective or consistent approach to outlier detection.

To conclude, the Agreement-Based Evaluation Metric offers a compact and interpretable means of assessing consistency across outlier selection methods. Quantifying how frequently the selected outliers of each method are corroborated by others provides an additional perspective on their relative performance, complementing previous analyses and offering further support for practical method selection.

## 5. Concluding Remarks

This article presented an analysis of outlier record selection techniques using an incremental dataset of caption-described images, approached in a non-standard manner. While the captions were treated as the primary data, the corresponding images served merely as visual references, offering a human-readable interpretation of the described content. The captions were tokenized into individual words (tags), which were then used as the basis for analysis. In some methods, unsupervised preprocessing techniques such as MDS were employed to better structure the data.

Experiments were conducted on both the original and preprocessed versions of the dataset to identify potential outlier records. Images selected using the original data served as a reference for comparison. In addition to this baseline, three types of methods were employed for outlier detection: metric and non-metric *MDS*, and the proposed *AOS* method—an agglomerative clustering-based approach to outlier selection. All methods were evaluated using both Euclidean and cosine distance metrics. For *AOS*, an additional categorical hyperparameter was introduced to determine the dendrogram cutoff strategy.

In each case, outliers were successfully identified based on caption information. Using the proposed evaluation metric, the methods were assessed by the degree to which their selected outliers overlapped, i.e., how frequently individual records were identified by more than one method. The highest agreement was observed for the *AOS* method with level cutoff and Euclidean distance. However, the metric *MDS*-based method achieved the best performance under the cosine distance metric. Overall, the combination of all approaches provided the most comprehensive and robust outlier detection performance.

In the case of high-dimensional data, rather than identifying outliers directly in the original feature space, one can first apply preprocessing techniques that transform the data into more tractable representations. In this study, machine learning methods such as hierarchical clustering and dimensionality reduction via the *MDS* method were used as preprocessing steps for caption-based image descriptions. The experimental results confirm the effectiveness of these transformations in enhancing outlier selection quality.

The outlier selection methods introduced and evaluated here are generalizable and applicable across a wide range of datasets. Depending on the vector space used to construct the distance matrix, the methods may highlight different types of outliers—those that are distinct under varying distance perspectives. To fairly evaluate and compare the results of each method, an Agreement-Based Evaluation Metric was introduced. This non-parametric metric measures the average degree of consensus among methods by quantifying how frequently the same records are selected as outliers. The influence of input data preprocessing and vector representation on such consensus remains an open question for future work.

## Figures and Tables

**Table 1:** Results of MDS calculations for subsets of 100, 200, 500, 1000, and 2000 records. The table shows the stress value of the best solution, the number of iterations, and the average total runtime with standard deviation—the algorithm was executed three times for each subset size to compute the averages.

Subset Size (Number of Records)	MDS Stress Value	Number of Iterations	Total Time [s]
100	507.82	679	3.6±1.2
200	2146.91	751	5.4±0.2
500	13,586.69	1274	54.9±1.1
1000	55,460.22	1364	230.3±0.4
2000	221,759.35	1457	1256.4±1.3

**Table 2:** Outlier IDs selected by different methods for a subset of 500 records. The IDs correspond to individual records in the dataset. For visual comparison across methods, identical outlier selections are highlighted using consistent colors and font styles—the same color or style indicates that the corresponding record was identified as an outlier by multiple methods. This visual encoding supports an intuitive cross-method comparison of detection consistency.

Method	Distance Metric	
	Euclidean	Cosine
AOS with level cutoff	2111, 6163, **862**, 5315, 1147, *5194*, **2178**, 1848, 5618, 1984, 3704	7507, 4482, 6954, 6130, 5419, 5114, 6291, 7014, 6672, **54**, 5336, 920, 6916, *5194*
AOS with last-*p* cutoff	854, *881*, 2111, 6163, **862**, 5315, 1147, *5194*, **2178**, 1848, 5618, 1984, 3704, 8451, 845	6291, 7014, 6672, 1984, 1322, 1897, 1896, 4437, 1147, 7833, 4560, 1564, 372, 7123, 177, *5194*
Metric MDS	7927, 2111, **2178**, 6163, *881*	1564, 7639, 920, **54**, 6916
Non-metric MDS	1728, 4198, 7916, 5197, 5725	
Real data based	1848, 5315, 2111, 6163, **862**	7507, 1984, **54**, 7317, *5194*

**Table 3:** Common outliers selected by specified methods. For comparison purposes, all results are shown on the subset with 500 records. For ease of representation, abbreviations are used: MME/MMC—Metric MDS with Euclidean/cosine distance; NM—Non-Metric MDS; RE/RC—Real data based, Euclidean/cosine distance; AEL/ACL—AOS with level cutoff, Euclidean/cosine distance; AEP/ACP—AOS with last-*p* cutoff, Euclidean/cosine distance. The font styles and colors correspond to those used in Table 2 to highlight outliers selected by multiple methods. Matching colors and styles identify the same outlier across methods, facilitating visual comparison of overlaps between method pairs.

	MME	NM	RE	MMC	RC	AEL	ACL	AEP	ACP
MME									
NM	-								
RE	6163, 2111	-							
MMC	-	-	-						
RC	-	-	-	**54**					
AEL	**2178**, 6163, 2111	-	5315, 6163, 1848, **862**, 2111	-	1984, *5194*				
ACL	-	-	-	920, 6916, **54**	*5194*, 7507, **54**	*5194*			
AEP	*881*, **2178**, 6163, 2111	-	5315, 6163, 1848, **862**, 2111	-	1984, *5194*	1984, **2178**, 5315, *5194*, 5618, 6163, 1848, 3704, 1147, **862**, 2111	*5194*		
ACP	-	-	-	1564	1984, *5194*	1984, *5194*, 1147	6672, *5194*, 6291, 7014	1984, *5194*, 1147	

**Table 4:** Number of outliers selected by different methods on the subset of 500 records. The values reflect how each method’s configuration—including distance metric and cutoff strategy—influences the number of identified outliers. Note that non-metric MDS and the real-data-based method always produce a fixed number of outliers, while the number for AOS depends on the structure of the dendrogram and the specific cutoff method applied.

Method	Distance Metric	
	**Euclidean**	**Cosine**
AOS with level cutoff	11	14
AOS with last-*p* cutoff	15	16
Metric MDS	5	5
Non-metric MDS	5	
Real data based	5	5

**Table 5:** Number of outliers commonly selected by each pair of methods. Bold indicates the pair with the largest number of commonly selected outliers. For comparison purposes, all results refer to the subset of 500 records. For ease of reference, abbreviations are used: MME/MMC—Metric MDS with Euclidean/cosine distance; NM—Non-Metric MDS; RE/RC—Real data based, Euclidean/cosine distance; AEL/ACL—AOS with level cutoff, Euclidean/cosine distance; AEP/ACP—AOS with last-*p* cutoff, Euclidean/cosine distance.

	MME	NM	RE	MMC	RC	AEL	ACL	AEP	ACP
MME									
NM	0								
RE	2	0							
MMC	0	0	0						
RC	0	0	0	1					
AEL	3	0	5	0	2				
ACL	0	0	0	3	3	1			
AEP	4	0	5	0	2	**11**	1		
ACP	0	0	0	1	2	3	4	3	

**Table 6:** Mean values of the Agreement-Based Evaluation Metric for Outlier Selection Methods (ABEM) calculated for each method. All results are based on the subset of 500 records. Methods are sorted in descending order by ABEM value to highlight stronger agreement with other methods.

Method	Distance Metric	Mean ABEM Value
Real data based	euclidean	3.400000
AOS with level cutoff	euclidean	3.272727
Real data based	cosine	3.000000
Metric MDS	euclidean	2.800000
AOS with last-*p* cutoff	euclidean	2.733333
Metric MDS	cosine	2.000000
AOS with level cutoff	cosine	1.857143
AOS with last-*p* cutoff	cosine	1.812500
Non-Metric MDS		1.800000

**Figure A1:**
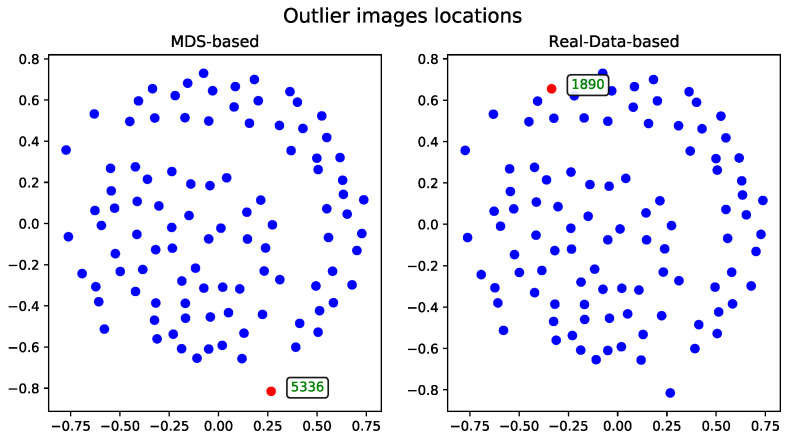
Outliers (marked with red) selected by metric MDS for a subset of 100 images using the cosine distance matrix. IDs correspond to records shown across all method visualizations.

**Figure A2:**
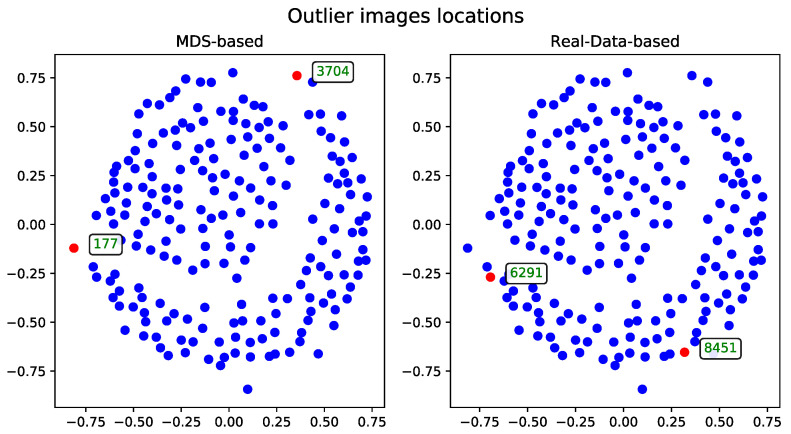
Outliers (marked with red) selected by metric MDS for a subset of 200 images using the cosine distance matrix.

**Figure A3:**
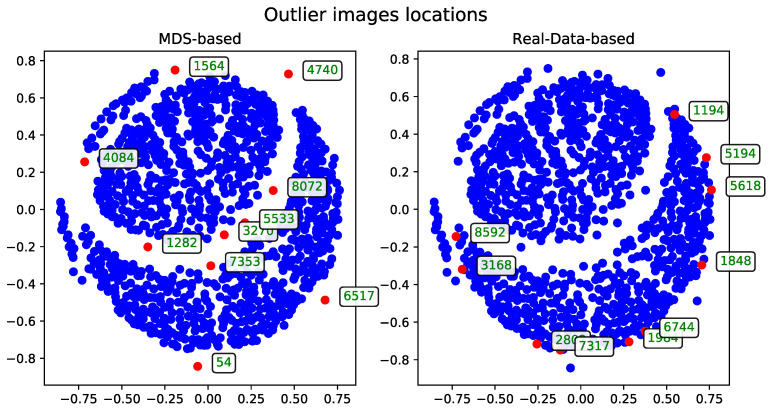
Outliers (marked with red) selected by metric MDS for a subset of 1000 images using the cosine distance matrix.

**Figure A4:**
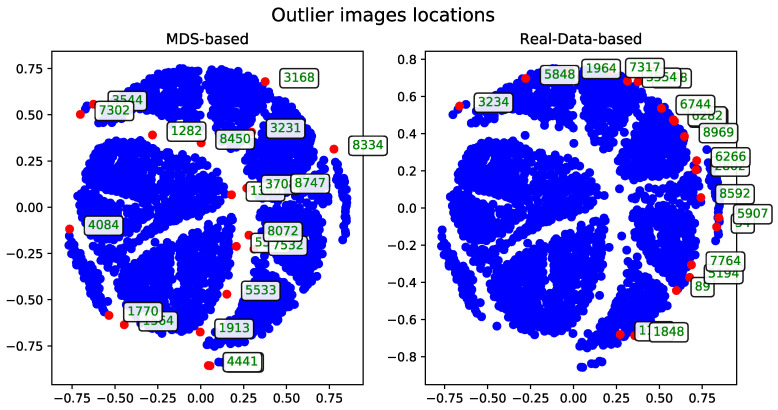
Outliers (marked with red) selected by metric MDS for a subset of 2000 images using the cosine distance matrix.

**Figure A5:**
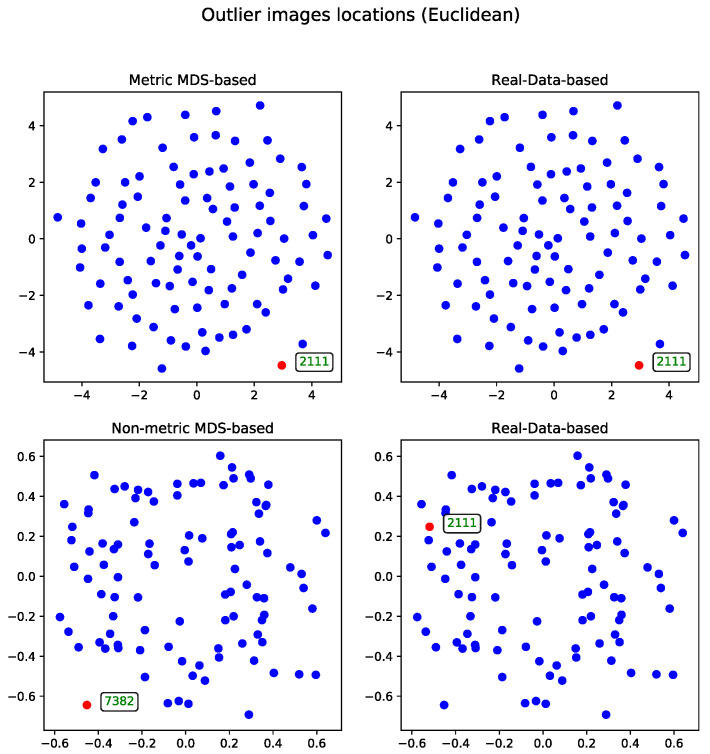
Comparison of outlier records (marked with red) selected by metric and non-metric MDS for a subset of 100 images using the Euclidean distance matrix.

**Figure A6:**
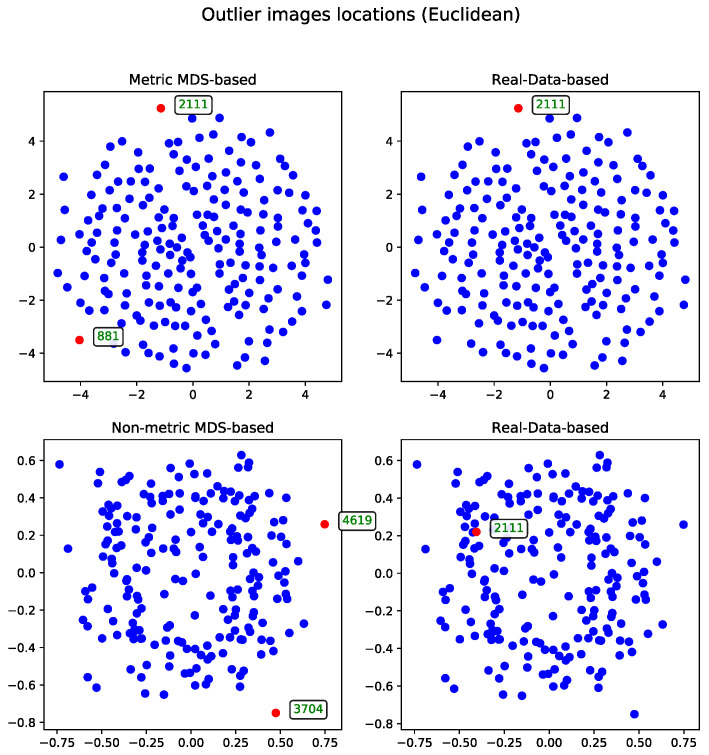
Comparison of outlier records (marked with red) selected by metric and non-metric MDS for a subset of 200 images using the Euclidean distance matrix.

**Figure A7:**
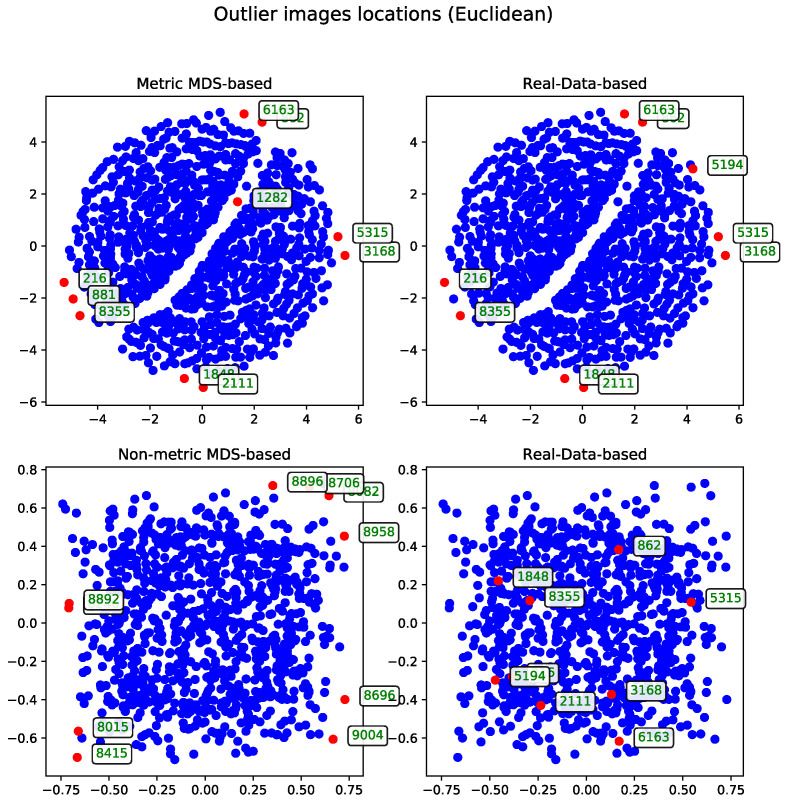
Comparison of outlier records (marked with red) selected by metric and non-metric MDS for a subset of 1000 images using the Euclidean distance matrix.

**Figure A8:**
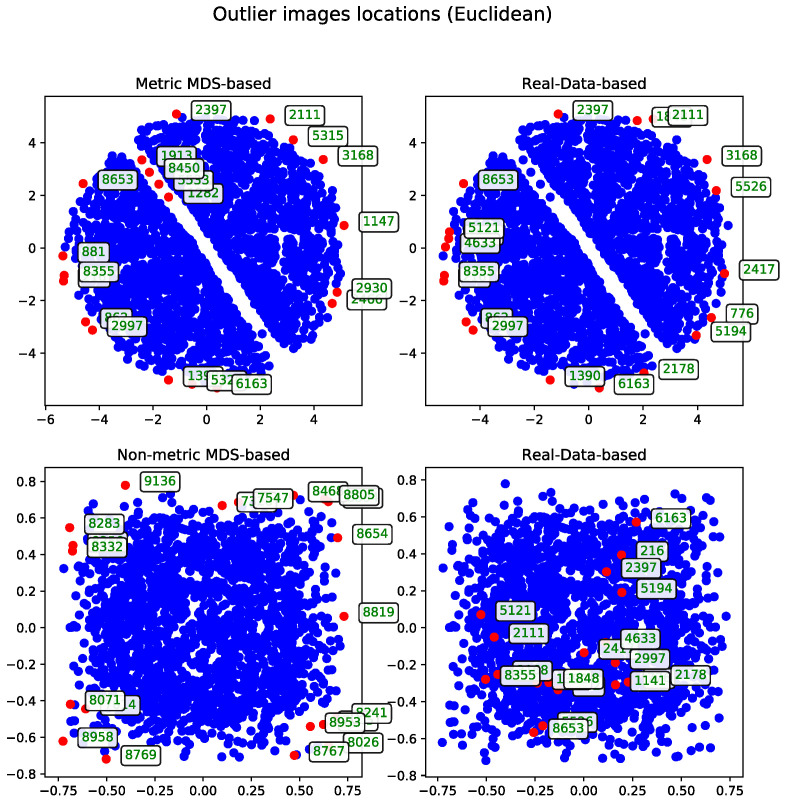
Comparison of outlier records (marked with red) selected by metric and non-metric MDS for a subset of 2000 images using the Euclidean distance matrix.

## Data Availability

The original contributions presented in this study are included in the article. Further inquiries can be directed to the corresponding author.

## Appendix A. Additional Figures for Other Subset Sizes

This appendix presents additional visualizations for various subset sizes—100, 200, 1000, and 2000 records—to supplement the analysis shown in Section 3.6.1. The plots follow the same methodology and visualization conventions as in the main part of the manuscript.

### Appendix A.1. Cosine Distance-Based MDS Visualizations

The figures below show the results of metric MDS with cosine distance matrix for different subset sizes. These complement the main analysis presented in Figure 1.

### Appendix A.2. Euclidean Distance-Based MDS Visualizations

The figures below show the results of both metric and non-metric MDS with Euclidean distance matrix, analogous to Figure 3 in the main text.
